# Preoperative prediction of intrahepatic cholangiocarcinoma lymph node metastasis by means of machine learning: a multicenter study in China

**DOI:** 10.1186/s12885-022-10025-4

**Published:** 2022-08-29

**Authors:** Tingfeng Huang, Hongzhi Liu, Zhaowang Lin, Jie Kong, Kongying Lin, Zhipeng Lin, Yifan Chen, Qizhu Lin, Weiping Zhou, Jingdong Li, Jiang-Tao Li, Yongyi Zeng

**Affiliations:** 1 Department of Hepatobiliary Surgery, Mengchao Hepatobiliary Hospital of Fujian Medical University, Xihong Road 312, Fuzhou, Fujian, 350025 PR China; 2 Department of Radiology, Mengchao Hepatobiliary Hospital of Fujian Medical University, Fuzhou, China; 3 Department of Hepatobiliary, Heze Municiple Hospital, Heze, China; 4 The Third Department of Hepatic Surgery, Eastern Hepatobiliary Surgery Hospital, Second Military Medical University, Shanghai, China; 5 Department of General Surgery, Institute of Hepatobiliary-Pancreatic-Intestinal Diseases, Affiliated Hospital of North Sichuan Medical College, Nanchong, China; 6 Department of Hepatobiliary and Pancreatic Surgery, The Second Affiliated Hospital of Zhejiang University School of Medicine, Hangzhou, China

**Keywords:** Intrahepatic cholangiocarcinoma, Lymph node metastasis, Machine learning

## Abstract

**Background:**

Hepatectomy is currently the most effective modality for the treatment of intrahepatic cholangiocarcinoma (ICC). The status of the lymph nodes directly affects the choice of surgical method and the formulation of postoperative treatment plans. Therefore, a preoperative judgment of lymph node status is of great significance for patients diagnosed with this condition. Previous prediction models mostly adopted logistic regression modeling, and few relevant studies applied random forests in the prediction of ICC lymph node metastasis (LNM).

**Methods:**

A total of 149 ICC patients who met clinical conditions were enrolled in the training group. Taking into account preoperative clinical data and imaging features, 21 indicators were included for analysis and modeling. Logistic regression was used to filter variables through multivariate analysis, and random forest regression was used to rank the importance of these variables through the use of algorithms. The model’s prediction accuracy was assessed by the concordance index (C-index) and calibration curve and validated with external data.

**Result:**

Multivariate analysis shows that Carcinoembryonic antigen (CEA), Carbohydrate antigen19-9 (CA19-9), and lymphadenopathy on imaging are independent risk factors for lymph node metastasis. The random forest algorithm identifies the top four risk factors as CEA, CA19-9, and lymphadenopathy on imaging and Aspartate Transaminase (AST). The predictive power of random forest is significantly better than the nomogram established by logistic regression in both the validation group and the training group (Area Under Curve reached 0.758 in the validation group).

**Conclusions:**

We constructed a random forest model for predicting lymph node metastasis that, compared with the traditional nomogram, has higher prediction accuracy and simultaneously plays an auxiliary role in imaging examinations.

## Introduction

Intrahepatic cholangiocarcinoma (ICC) is an uncommon but lethal disease that originates from bile duct epithelial cells above the secondary bile duct branch [1]. This condition highly malignant and has a poor prognosis [2] and its global incidence has been reported to be on the rise [3–5]. About 35% of the patients with ICC already had lymph node metastasis (LNM) at the time of diagnosis [6]. An international multicenter study shows that LNM in cases of ICC is associated with an adverse effect on survival rates [7]. Therefore, exploring a new and effective method to evaluate the status of lymph node metastasis is of great significance for the diagnosis and treatment of patients with ICC [8–10].

Clinicians who rely solely on the imaging data to judge whether there is lymph node metastasis or not are susceptible to missed or inaccurate diagnosis [11]. Therefore, it is particularly important to comprehensively evaluate the status of the lymph nodes to better understand a patient’s condition. In the era of rapid development of medical technology and diversified treatment options, the demand for methods to accurately judge the conditions of lymph nodes is increasing every year. Traditionally, logistic regression analysis and nomogram have been widely used in building prognostic predictive models [13, 14]. Some variables such as CA19-9, CEA, lymph node size on imaging (CT/MRI) (≥ 1 cm) are commonly extracted in the modeling process. However, the linearity assumption cannot model the complex, multidimensional and nonlinear relationship between variables, so these approaches have several limitations. Recently, machine learning has become one of the noteworthy directions of prediction models [15]. It uses nonlinear functions and takes into account the interaction between variables, thus improving prediction accuracy [16]. Currently, machine-learning techniques are rarely utilized in predicting LNM of ICC and many studies that applied this method were limited with relatively small sample sizes and lack of external validation dataset. Improved predictive ability could be achieved by applying traditional statistical and machine learning methods. An increasing number of studies have been paying attention to the limitations and advantages of traditional methods and machine learning and how they interfere with each other.

There are many machine learning methods available in the research community and random forest is one of the most used ones in clinical classification issues [17]. Random forests operate by constructing a multitude of decision trees. In the training process, the interaction between each variable can be detected and their importance can be sorted. In order to use preoperative indicators, including many complex variables, we used machine learning and logical regression in the modeling process. Relevant literature shows that patients with hepatitis B virus (HBV) infection tend to have a lower rate of lymph node metastasis [18, 19]. However, the P value of HBV was less than 0.05 in the multivariate analysis, so we did not include it in our modeling.

The purpose of this study is to add hepatitis as a predictor and to develop a model to accurately predict ICC LNM before surgery. The differences between machine learning and line diagram were also compared, and the credibility of the model was verified by external data.

## Materials and methods

### Patients of the training cohort

This study was approved by the Ethics Committee of the Mengchao Hepatobiliary Hospital of Fujian Medical University and exempts the requirement of written informed consent (IRB No 2019_049_01). All procedures were performed in accordance with the World Medical Association Declaration of Helsinki. The database was retrospectively derived from patients with pathologically confirmed ICC who underwent hepatic resection and lymph node dissection at the Eastern Hepatobiliary Surgery Hospital, Second Military Medical University (EHSH) (*n* = 149, from Jan. 2013 to Sept. 2018). We validated our model with an external data set consisting of three cohorts of patients from Mengchao Hepatobiliary Hospital of Fujian Medical University, The Second Affiliated Hospital Zhejiang University School of Medicine, and the Affiliated Hospital of North Sichuan Medical College (*n* = 62).

The inclusion criteria were as follows: (1) pathological confirmation of ICC, (2) no intrahepatic and extrahepatic metastases, (3) Child–Pugh A/B before surgery, (4) underwent lymph node dissection. We excluded patients who: (1) did not undergo lymph node dissection or palliative surgery, (2) died within 30 days after surgery, (3) had incomplete preoperative imaging or serological data. (4) underwent neoadjuvant chemotherapy. Qualified patients from Eastern Hepatobiliary Surgery Hospital between 2013 and 2018 were placed in the training group, whereas patients who met the conditions in the other three hospitals between 2016 and 2020 were used for external verification.

### Clinicopathologic variables

The serological data we used were the results of the most recent test conducted two weeks before the operation. The preoperative imaging data were obtained from contrast-enhanced Computed Tomography or contrast-enhanced Magnetic Resonance Imaging (CT/MRI) prior to surgery. Based on our clinical experience, the variables included sex, age, hepatitis B, tumor size on imaging (MRI/CT), number of tumors on imaging (single or multiple), liver cirrhosis on imaging, enlargement of the lymph nodes on CT/MRI imaging, total bilirubin, ALT, GGT, AST/ALT (≤ 1, > 1), ALP, PT, white blood cell count (WBC), platelets (PLT), red blood cell (RBC), alpha-fetoprotein (AFP) (≤ 20 ng/ml, > 20 ng/ml), carcinoembryonic antigen (CEA) (≤ 10 ng/ml, > 10 ng/ml), and carbohydrate antigen 19–9 (CA19-9) (≤ 39 ng/ml, > 200u/ml).Furthermore, the maximum tumor diameter on imaging was defined as the biggest diameter. Solitary tumor was defined as only one tumor lesion in the liver, and two or more cancerous lesions were defined as multifocality. Enlargement of the lymph nodes was defined as > 1 cm on CT/MRI imaging.

### Statistical analysis

All data analyses were performed by R software (version4.1.0, http://www.r-project.org). The Mann–Whitney U test was used to compare continuous variables, and chi-square or Fisher's exact test were used to compare categorical variables. C-Index was used to evaluate the forecasting effect of the model. All *P* values were based on a two-sided statistical analysis. *P* < 0.05 was considered statistically significant in single factor and multifactor analyses, and the variables with *p* ≤ 0.05 were used to establish the prediction model. A small amount of the missing sample was supplemented by the mean imputation. C-Index is mainly used to reflect the discrimination ability of various forecasting models, and to investigate whether the model is correct in forecasting. In this research, The C-Index was calculated using the Hmisc (4.7) package of R language(4.1.3).

### Surgical strategy

Hepatectomy was performed in patients who met the following criteria: 1) diagnosed with a technically resectable tumor with no evidence of extrahepatic metastases, and 2) generally good condition with healthy liver functions and adequate remaining liver volume. LND (lymph nodes dissection) was performed if 1) the patient was most likely to have LNM as determined by the preoperative multidisciplinary team, and 2) had enlarged lymph nodes that were manually detected by the surgeon during the procedure. The methods included subtotal hepatectomy and small hepatectomy. According to Couinaud's classification, major hepatectomy is defined as the removal of three or more liver segments, and resection of less than three liver segments is defined as minor hepatectomy. The procedure of LND included skeletonization of the hepatoduodenal ligament and resection of para hepatic artery lymph nodes at least to the second station, which was a little different from each center.

## Results

### Baseline characteristics of patients

The database was retrospectively derived from patients with pathologically confirmed ICC who underwent hepatic resection and lymph node dissection at Eastern Hepatobiliary Surgery Hospital, Second Military Medical University (EHSH) (*n* = 149). We validated our model with an external data set consisting of three cohorts of patients from Mengchao Hepatobiliary Hospital of Fujian Medical University, The Second Affiliated Hospital Zhejiang University School of Medicine, Affiliated Hospital of North Sichuan Medical College (*n* = 62).

Patient baseline demographic and clinical data are shown in Table 1. There were no significant differences in the percentage of LNM between the training group and the validation group (*P* = 0.944). The median patient age was 56.0 years (IQR 47.0–62.0) in the training cohort, and the median patient age was 60.0 years (IQR 52.0–64.0) in the validation cohort. Most patients were male (*n* = 77, 51.7%), and the majority of patients had a single lesion (*n* = 134, 90%) with a median tumor size of 6.2 cm (IQR 4.8–8.0) in the training group. Similar results were also observed in validation groups, and no statistical difference was detected.

### Construction of the model

#### Variables

**Fig. 1:**
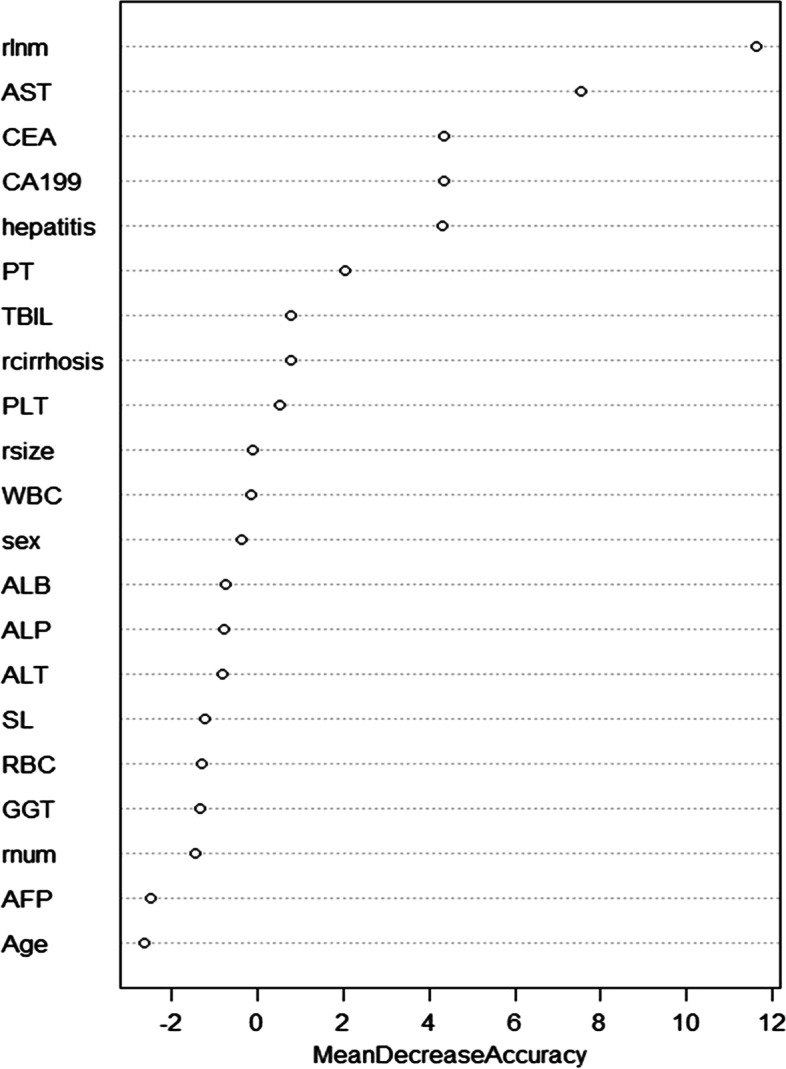
The variable importance by random forest

The following univariate and multivariate logistic regression analysis showed potential independent risk factors, including CEA, CA19-9, and enlargement of the lymph nodes on CT/MRI imaging (Table 2). The importance ranking of factors calculated by random forest is shown in Fig. 1. The importance ranking of variables related to lymph node metastasis was obtained according to the Gini index algorithm of the importance of random forest variables. The top four variables were: lymph node metastases on imaging, AST, CEA > 10 ng/ml, and CA19-9 > 39 ng/ml.

**Table 1:**
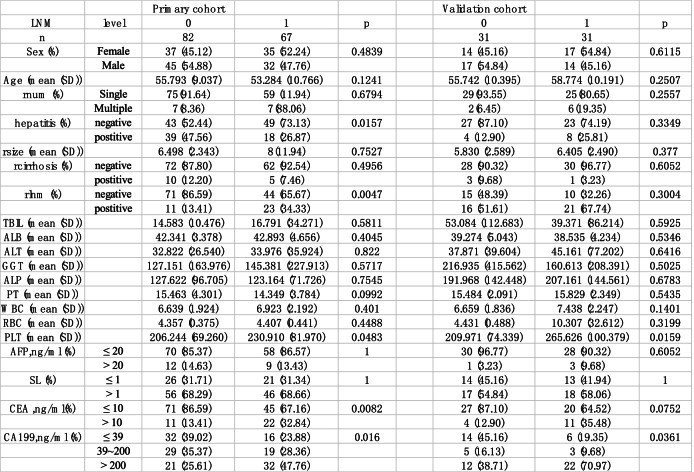
Characteristics of patients in the primary and validation cohorts

*O* negative, *1* Positive, *Sex: 0* female *1* male, *LNM* Pathology confirmed lymphnode metastasis, *rnum* Number of tumors on imaging (single or multiple), *hepatitis* Hepatitis B or not, *TBIL* Total Bilirubin, *ALB* album in, *ALT* alanine amino transferase, *PT* prothrombintime, *WBC* leukocyte, *RBC* erythrocyte, *PLT* platelet, *AFB* Alpha fetoprotein, *SL* AST/ALT, *CEA* Carcinoembryonic antigen, *CA199* Carbohydrate antigen 199, *ALP* Alpha fetoprotein

**Table 2:**
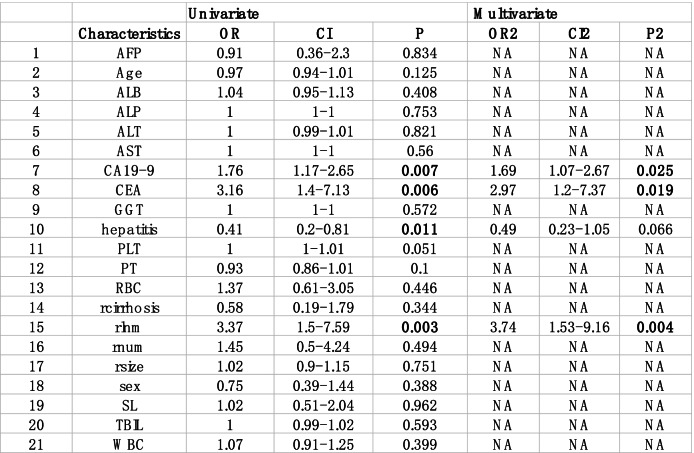
Univariate and multivariate logistic regression analysis

Abbreviations: *AFP* Alpha fetoprotein, *ALB* albumin, *ALP* Alpha fetoprotein, *ALT* Alanine aminotransferase, *AST* Aspartate Transaminase, *CA19-9* Carbohydrate antigen 19–9, *CEA* Carcinoembryonic antigen, *GGT* γ-glutamyl transferase, *hepatitis* Hepatitis B positive, *PLT* platelet, *PT* Prothrombin time, *RBC* erythrocyte, *rcirrhosis* Imaging hepatic cirrhosis, *rlnm* lymph node metastasis on imaging, *rnum* multiple tumors on imaging, *rsize* Imaging tumor size, *SL* AST/ALT, *TBIL* Total bilirubin, *WBC* leukocyte

#### Establishment and verification of the predictive nomogram

**Fig. 2:**
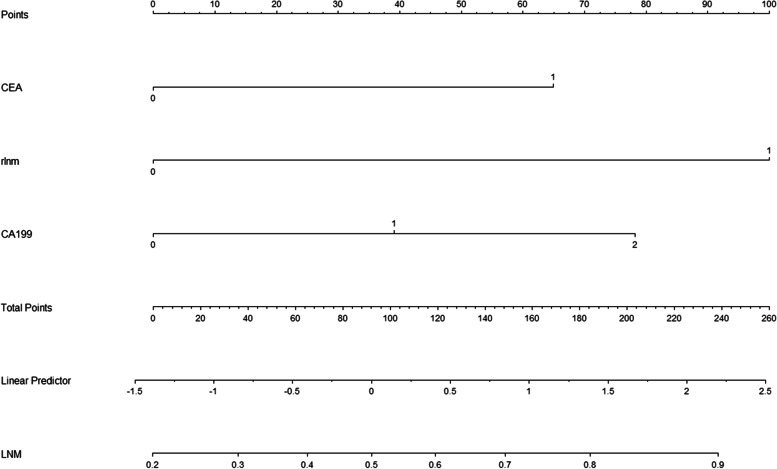
The nomogram of preoperative prediction model for LNM of ICC

**Fig. 3:**
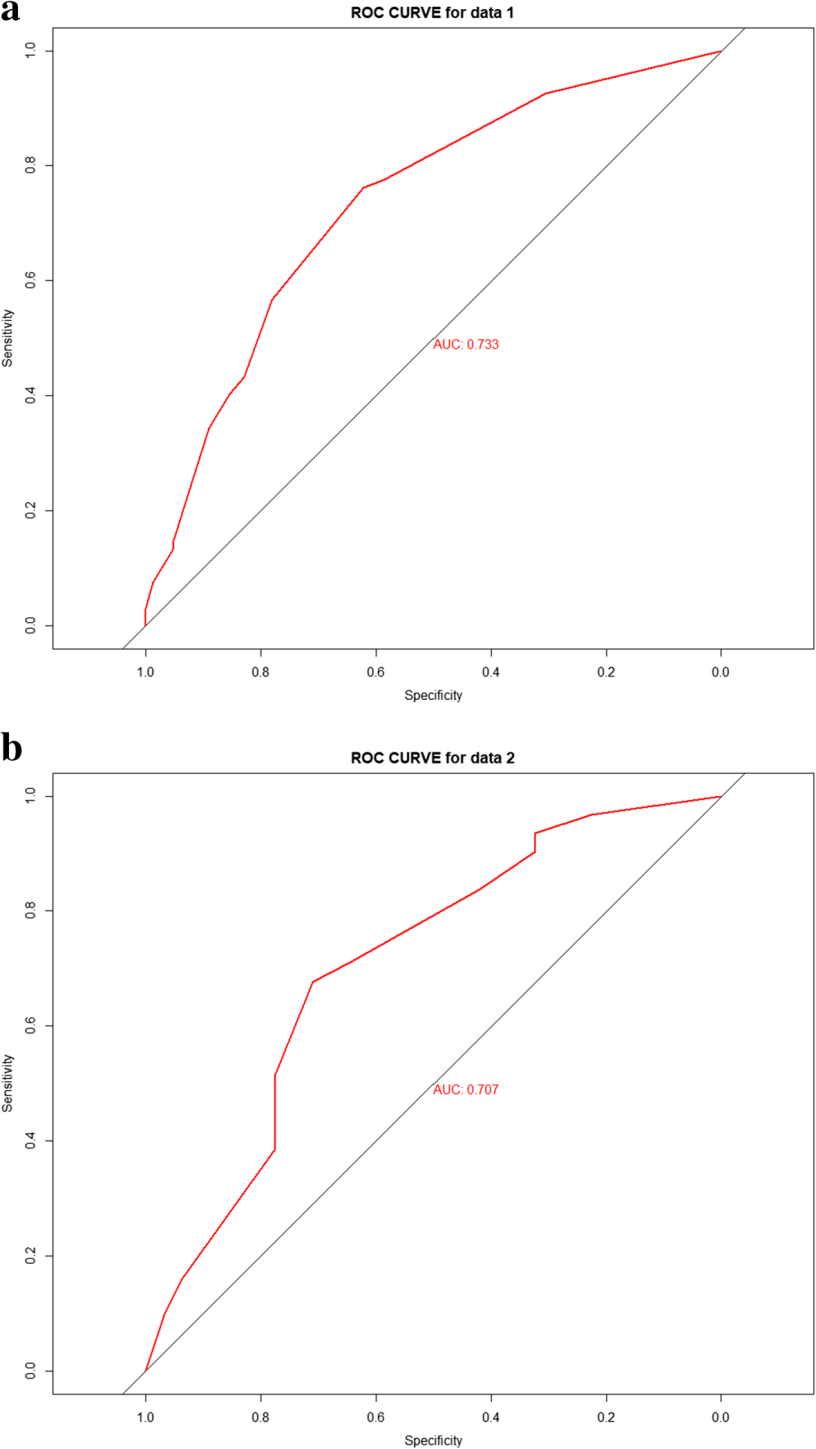
**a** The C-index of nomogram in training group. **b** The C-index of nomogram in validation group

**Fig. 4:**
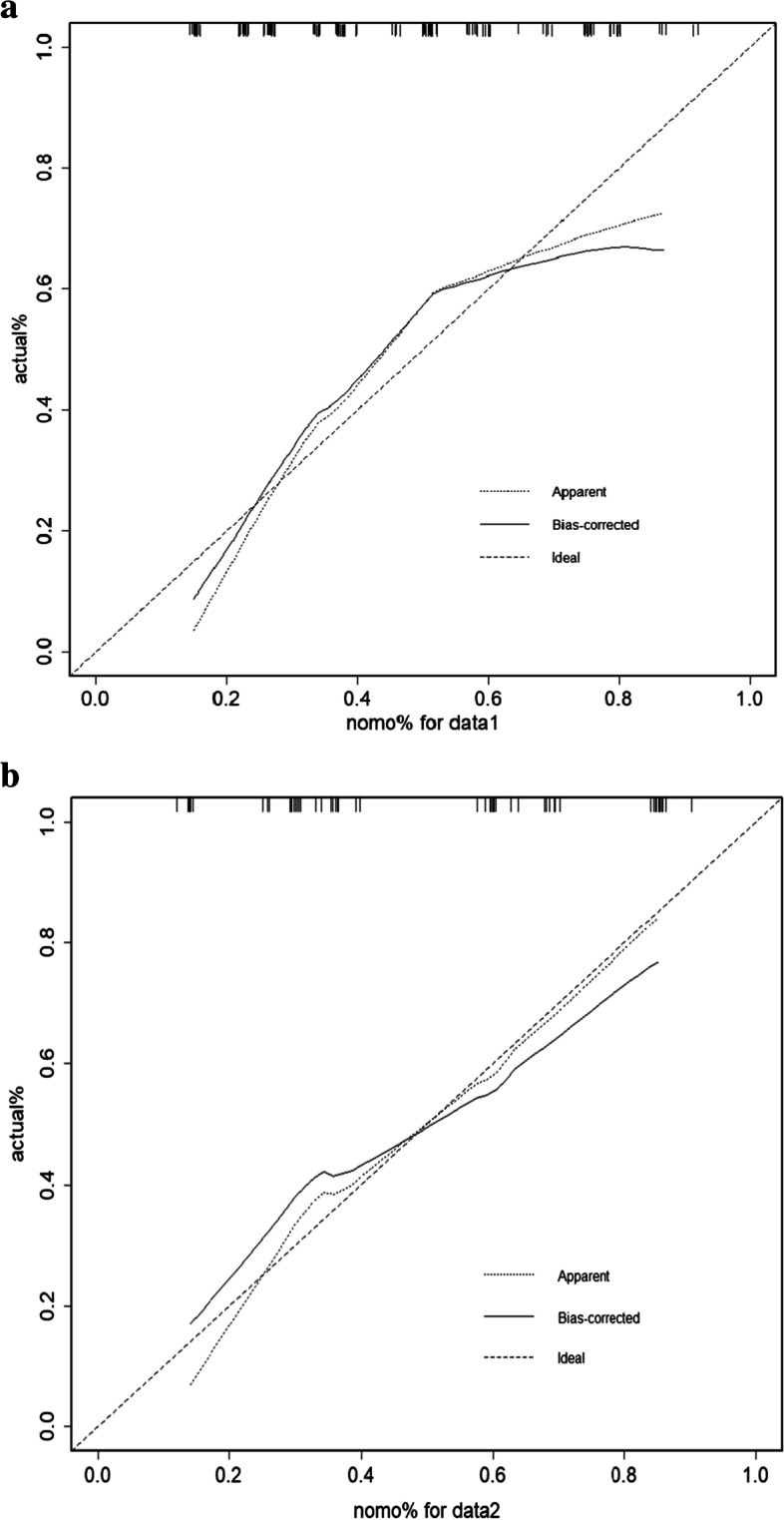
**a** The calibration curve of train group. **b** The calibration curve of test group

The results of the aforementioned multivariate logistic regression analysis showed that elevated CA19-9 levels (> 200u/ml, *P* = 0.019), elevated CEA (> 10 ng/ml, *P* = 0.025) and enlargement of the lymph nodes (> 0.1 cm, *P* = 0.004) were independent postoperative risk factors to construct the nomogram (Fig. 2). In the nomogram, the value for each patient is located on each variable axis, and a line is drawn upward to determine the number of points received for each variable value. The sum of these numbers is located on the total score axis, and a line is drawn down to the outcome axis to determine the likelihood of developing lymph node metastases. The C-index was 0.733 (95% CI:0.654–0.813) for the prediction nomogram in the training group (Fig. 3a). In the validation group, the C-index was 0.707 (95% CI: 0.576–0.837) for the prediction of LNM (Fig. 3b). In the logistic regression test group, the Sensitivity is 0.72, the Specificity is 0.59, the positive predictive value is 0.42, the negative predictive value is 0.84, the false positive is 0.60, the false negative is 0.28, the Accuracy is 0.63 (95%CI:0.50–0.75). In the logistic regression train group, the Sensitivity is 0.69, the Specificity is 0.68, the positive predictive value is 0.78, the negative predictive value is 0.57, the false positive is 0.32, the false negative is 0.31, the Accuracy is 0.68 (95%CI:0.60–0.76). The calibration curve is illustrated in Fig. 4A/B. The curve shows that the occurrence of lymph node metastasis predicted before the operation was in good agreement with the actual situation in both the prediction and the validation groups.

#### Development and validation of the random forest

**Fig. 5:**
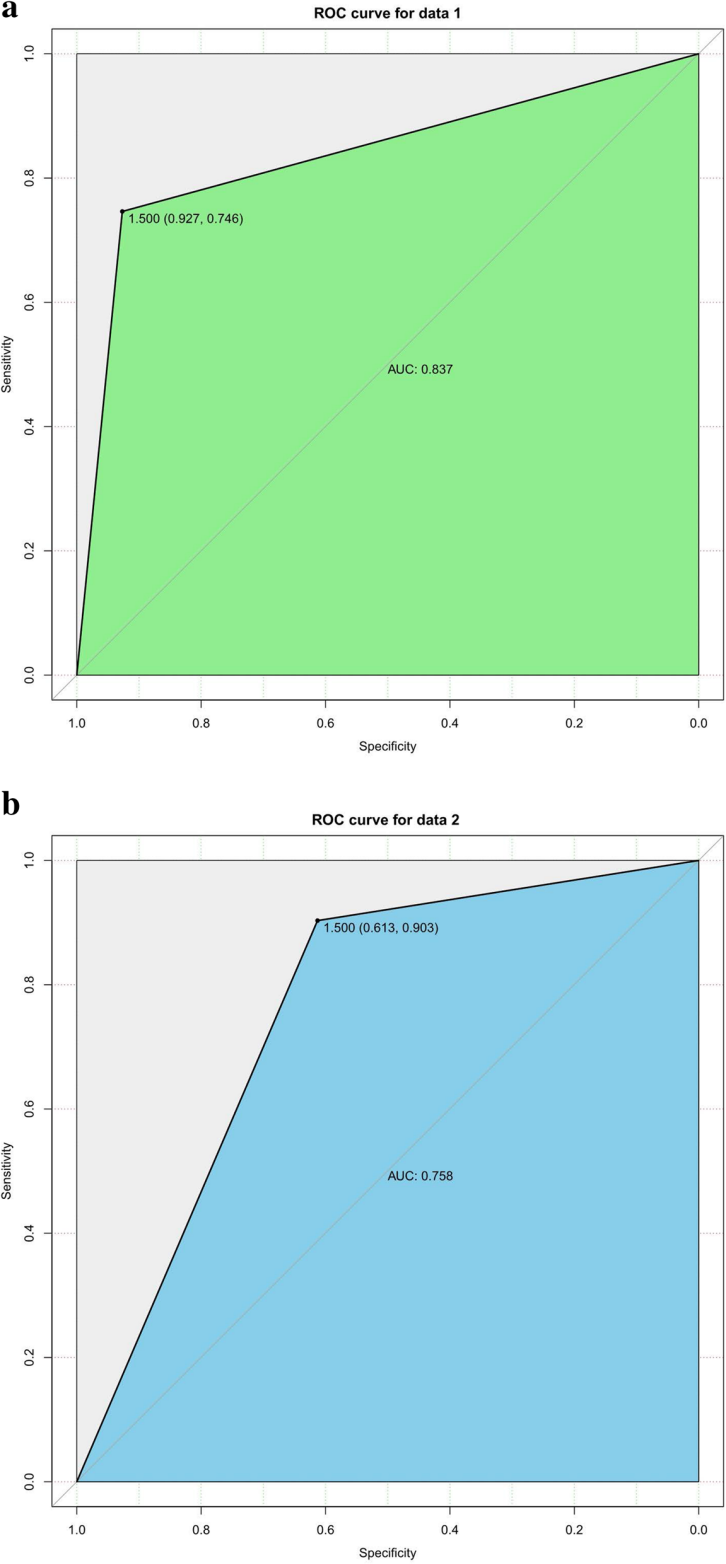
**a** The C-index of random forest in training group. **b** The C-index of random forest in validation group

We constructed random forest classifiers consisting of 500 classification and regression trees (CART), using the implementation in the Random forest R package. The prediction factors were incorporated into the training by the integrated algorithm, the importance of variables was ranked according to the algorithm, and then a stochastic forest random model was constructed. The C-index was 0.837(95%CI: 0.777–0.896) for the random forest in the training group (Fig. 5a). In the validation cohort, the C-index was 0.758 (95% CI: 0656–0.860) for the prediction of LNM (Fig. 5b). In the Random forest train group, the Sensitivity is 0.82, the Specificity is 0.90, the positive predictive value is 0.93, the negative predictive value is 0.75, the false positive is 0.41, the false negative is 0.28, the Accuracy is 0.85 (95%CI:0.78–0.90). In the Random forest test group, the Sensitivity is 0.86, the Specificity is 0.70, the positive predictive value is 0.61, the negative predictive value is 0.90, the false positive is 0.11, the false negative is 0.19, the Accuracy is 0.76 (95%CI:0.63–0.86).

## Discussion

Intrahepatic cholangiocarcinoma (ICC) is a rare and fatal disease [2, 5], and lymph node metastasis associated with this condition is an independent risk factor that affects the prognosis of ICC patients [20], and the choice of surgical methods for treatment [21]. The vast majority of related studies in this realm used logistic regression to screen risk factors and then established nomograms to try to predict lymph node metastasis [22, 23]. However, most studies lacked validation with multicenter data, and only a few researchers have applied random forest to predict lymph node metastasis in patients with ICC. To the best of our knowledge, we are the first group to use random forest to predict ICC lymph node metastases, compare the method with traditional techniques and validate the prediction accuracy with external data.

Multivariate logistic regression is widely used to construct nomograms. It removes confounding variables and helps better visualize the results. However, logistic regression can only establish a linear model, and too many variables can easily affect the model fitting and equation stability [23]. Machine learning is a versatile tool for data analysis that has been widely used in predicting other carcinomas [25–27]. The calculation process of random forest is a black box, but its accuracy is high, its operational speed is fast, and the calculations do not easily overfit [24]. However, it is important to acknowledge that this method may have specific limitations in the case of small sample sizes [28]. In this paper, a logistic regression model and a random forest model were established simultaneously, and the accuracy of the two methods was further compared.

Our nomogram was established based on important risk factors for lymph node metastasis screened through logistic regression, including elevated CA19-9 (greater than 200u/ml *P* = 0.019), elevated CEA (greater than 10 ng/ml *P* = 0.025), and lymphadenopathy on imaging (largest lymph node diameter greater than 0.1 cm *P* = 0.004). Previous studies have confirmed that these are important risk factors for lymph node metastasis in ICC [29–31]. It has also been reported that D-dimer combined with CA19-9 can predict lymph node metastasis in ICC [32]. Other scholars have also created nomograms established by logistic regression, and their results showed that they had good predictive power [12–14]. However, logistic regression is considered a limited method because it is difficult to further improve its prediction accuracy.

Recently, machine learning has been widely used in different research fields. Therefore, we innovatively adopted Random forest algorithm for LNM prediction in ICC. In our study, the Random forest showed a huge advantage and better prediction accuracy over traditional nomograms. Through random forest, we also acquired preoperative serological indicators that are highly correlated with lymph node metastasis, such as AST, CEA, and CA19-9. Taking CA19-9 as an example, some scholars believe that para-aortic lymph node metastases are already systemic metastases [33]. Preoperative CA19-9 levels have the potential to predict para-aortic lymph node metastasis, especially when CA19-9 levels are higher than 200 U/ml [31]. Furthermore, it has been reported that elevated AST in other tumors may be associated with lymph node metastasis [34]. This correlation may be linked with elevated AST occurring under conditions that lead to a higher proliferative state, tissue damage, and increased tumor cell renewal [35]. So, serological indicators combined with preoperative imaging can help us better determine the status of lymph nodes.

Nevertheless, it is important to acknowledge the limitation of our research. Considering that the accuracy of random forest, if the number of cases is increased further, random forest regression would be a method that is more persuasive and superior in quality. Also, if the sample size increases, the model will reducing the risk of overfitting. Furthermore, although each center involved in this study is a large Grade 3A hospital (3A hospitals are top-class hospitals in China), there may be some differences in the diagnosis and treatment of ICC patients among different medical institutions. However, the multicenter data is more representative and increases the applicability of the prediction model. At last, although relevant data from different centers in China were included for analysis, we still lacked data from international centers. Therefore, in the future, if the sample size can be expanded and data from large scale international centers can be included, our machine learning method can be improved to make more accurate predictions. Previous studies have shown that ICC patients with lymph node metastases tend to have poor survival rates [7, 20]. However, due to a lack of follow-up data, a detailed survival analysis of predicted patients could not be performed in this study. This informational gap can be solved if prospective studies are carried out in the future. At the same time, patients with hilar invasion tend to have lymph node metastasis [36], but due to the limitations of our data, this part of the content has not been studied in depth. We hope that we can further improve the relevant content in the future.

In summary, this study proposes a preoperative model for predicting LNM of ICC patients using random forest, a commonly used machine learning method. Our research design proved that this method is effective in distinguishing lymph node status. This research presents a novel approach, as this is the first time the random forest method is used to predict ICC lymph node metastasis. Compared with traditional methods, the random forest technique can trace more accurate predictions, which is why it is considered advantageous for complex medical studies like ours. The use of multicenter data made our research more representative and our results more reliable and applicable to other contexts. Through the double verification process of the nomogram and the random forest, patients can accurately judge the status of their lymph nodes before the operation and have access to essential data to guide their surgical treatments.

## Data Availability

All data included in this study are available upon request by contact with the corresponding author.
